# Seminal Calbindin 2 in Infertile Men With Varicocele: A Prospective Comparative Study

**DOI:** 10.1007/s43032-023-01237-5

**Published:** 2023-04-17

**Authors:** Sameh Fayek GamalEl Din, Ashraf Zeidan, Mohamed Ahmed Abdel Salam, Noha Abd EL Hafeez Abd El Kader, Sara Ahmed Mohamed, Mohamed Farag Azmy

**Affiliations:** 1 Department of Andrology, Sexology and STDs, Kasr Al-Ainy Faculty of Medicine – Cairo University, Cairo, Egypt; 2 Department of Clinical and Chemical Pathology, Faculty of Medicine, Beni-Suef University, Beni-Suef, Egypt; 3 Egypt ministry of health & population, Cairo, Egypt; 4 Department of Andrology, Sexology and STDs, Faculty of Medicine - Beni-Suef University, Beni-Suef, Egypt

**Keywords:** Varicocele, Oligoasthenoteratozoospermia, Seminal plasma Calbindin 2 (Calretinin and 29 kDa Calbindin)

## Abstract

The present study compared seminal calbindin 2 (CALB 2) levels and semen parameters in men with and without varicocele. CALB 2 is also known as calretinin and 29 kDa calbindin. The study was a case-control study conducted from April (2021) to March (2022) in the andrology department at Beni-Suef University hospital. The study included four matched groups: group (I) were controls (fertile normozoospermic men without varicocele) (*n*=24). Group (II) were fertile normozoospermic men with varicocele (*n*=24). Group (III) were infertile oligoasthenoteratozoospermia (OAT) men without varicocele (*n*=24). Group (IV) were infertile OAT men with varicocele (*n*=24). The lowest levels of seminal CALB 2 were found in patients with severe oligozoospermia which showed a statistically significant difference when compared to seminal CALB 2 in patients with normal, mildly low, or moderately low sperm counts. There were significant negative correlations between sperm concentration, sperm motility and percentage of normal sperm forms and seminal CALB 2. Seminal plasma CALB 2 may play a role in the negative impact of varicocele on the semen parameters especially sperm concentration, sperm motility and percentage of sperm normal forms. Future studies are needed to verify these findings.

## Introduction

Varicocele is defined as an abnormal venous dilatation and/or tortuosity of the pampiniform plexus in the scrotum [1]. Clinical varicocele is found in approximately 15% - 20 % of the normal adult male population and 35% - 44% of men with primary infertility [2–6]. Varicocele is frequently associated with low sperm count, decreased sperm motility and increased sperm abnormal morphology [7–9]. A study conducted on 716 patients with primary infertility and various grades of varicocele demonstrated increasing deterioration in semen parameters with increasing grade of varicocele [10]. Several theories have been raised to explain the mechanisms by which varicocele impairs male fertility. They included scrotal hyperthermia, retrograde flow of metabolites, Leydig cell dysfunction, hypoxia and impaired testicular artery perfusion [11].

Ca2+ binds thousands of proteins, affecting protein localization and function and an extensive number of cell signalling pathways [12, 13]. CALB 2 (Calretinin and 29 kDa Calbindin) is a Ca 2+-binding protein with a typical EF-hand calcium binding domain that can be reversibly combined with Ca 2+ as a calcium receptor. CALB 2 belongs to the calmodulin superfamily of Ca2+-binding proteins and was first discovered by Rogers in the retina of a chicken [14]. CALB 2 is primarily expressed in specific neurons, however, CALB 2 is also expressed in other tissues during development where it likely plays a role in proliferation and possibly differentiation [15]. CALB 2 is also expressed in both human and rat Leydig cells [16, 17] where it may play a role in testosterone biosynthesis: activation of phospholipase C induces Ca2+ influx into the cytoplasm [18], which in turn activates protein kinase C [19, 20], and PKC plays an important role in testosterone biosynthesis [21]. CALB 2 is also expressed in rat and mouse spermatozoa and CALB 2 knockout mice have impaired fertility [22]. These data suggest that CALB 2 expression is linked to spermatogenesis. Notably, CALB 2 was detected in immature Sertoli cells in testicular biopsies of men with non-obstructive azoospermia, however, the link between CALB 2 and defective development of functional Sertoli cells remains to be elucidated [23].

The WHO laboratory manual for the examination and processing of human semen (fifth edition, 2010) defines oligoasthenoteratozoospermia (OAT) as follows: the total number of the spermatozoa in the ejaculate should be below the lower reference limit, percentage of progressively motile spermatozoa below the lower reference limit and percentage of morphologically normal spermatozoa below the lower reference limit [24].

The present study was performed to begin to address the relationship between seminal CALB 2 and varicocele and male infertility.

## Patients and methods

### Study population

The present study was a case-control study conducted from April (2021) to March (2022) in the andrology department at Beni-Suef University hospital. The study was approved by the ethical committee of the Faculty of Medicine, Beni-Suef University on 9th May 2021 (FMBUREC/09052021) which conforms to Helsinki declaration (2013) [25]. Informed written consent was obtained from all participants before recruitment in the study after explaining the objectives of the work. Sample size calculation was done using the results of a recent study designed to detect seminal CALB 2 expression [16]. We calculated that the effect size was 0.848 (large effect size) and the alpha error was 0.05, and we used a power of 0.95. Accordingly, sample size was calculated with G*Power (3.1.9.4) software using a priori analysis for difference between two independent means (two groups). A total sample size of 94 participants was estimated for 90% power, α- error probability 0.05 and 10% dropout rate during follow up. Accordingly, the study included four matched groups: group (I) were fertile normozoospermic men without varicocele (controls) (*n*=24). Group (II) were fertile normozoospermic men with varicocele (*n*=24). Group (III) were infertile OAT men without varicocele (*n*=24). Group (IV) were infertile OAT men with varicocele (*n*=24).

### Inclusion criteria of the patients

Fertile males suffering from varicocele and infertile OAT males with and without varicocele.

### Exclusion criteria of the patients

Patients with azoospermia, smoking, leukocytospermia and abnormal Karyotyping were excluded.

All patients and healthy controls were subjected to the following:

Detailed histories were obtained from all participants. General and clinical examinations were performed. Varicocele was assessed with scrotal duplex. Semen analysis was analyzed according to WHO guidelines (fifth edition, 2010) [24].

Measurement of seminal CALB 2 concentrations:

CALB 2 level in seminal samples were assayed using an Human Calretinin ELISA kits supplied by SinoGeneclon Biotech Co., Ltd. Hangzhou , China, Cat. No.: SG-00383 was carried out according to the manufacturer’s instructions. This assay has high sensitivity and excellent specificity for detection of CALB 2. No significant cross-reactivity or interference between CALB 2 and analogues was observed. Sensitivity: 2.045 ng/ml. Assay range: 3 ng/ml → 600 ng/ml, intra-Assay: CV< 9% and inter-Assay: CV< 11%.

### Statistical analysis

The collected data were coded then entered and analyzed using the SPSS version 25 (Statistical package for social science) for windows 10. Descriptive analysis of the results was done in the form of percentage distribution for qualitative data and minimum, maximum, mean and standard deviation calculations for quantitative data. Cross tabulation and Chi Square test (χ2) were used for comparison between categorical variables and percentage values. Student t-test was used for comparison between means of two unrelated groups with a normal distribution. One way ANOVA test was used for comparison between means of more than two unrelated groups with a normal distribution. Pearson’s correlation analysis was done to evaluate linear relationship between CALB 2 expression and other semen parameters. Correlation graphs were drawn only for significant correlations, p<0.05. Correlations were considered positive (direct correlation) when r (correlation coefficient) had a plus signal and negative (inverse correlation) when r had a minus signal. Correlations were evaluated as weak when the absolute value of r = 0 – 0.35, moderate when r = 0.35 – 0.65, and strong when r > 0.65.

## Results

Table 1 lists the age and semen parameters of the study participants. The age of the patients was significantly higher in the infertile OAT without varicocele group compared to the other three groups. Sperm concentration and sperm motility were significantly higher in both of the fertile groups compared to the two infertile groups. CALB 2 level was significantly lower in the infertile OAT with varicocele group compared to the OAT group without varicocele and the normozoospermic group with varicocele and the control group. There were no significant differences in semen parameters between the fertile normozoospermic group and the control group.

**Table 1:** Shows the age and the characteristics of the semen parameters of the participants

	Controls (Fertile normozoospermic)	Cases		
		Infertile OAT without varicocele	Infertile OAT with varicocele	Fertile normozoospermic with varicocele
	Mean±SD	Mean±SD	Mean±SD	Mean ±SD
Age (years)	26.5±3.8	32.14±5.9**^,##,&&^	28.70±4.5	28.87±5.9
Semen volume (ml)	4.06±1.3	4.63±6.4	3.42±1.1	3.35±0.9
Sperm concentration (10^6^/ml)	49.32±16.5	13.42±15.5***^,&&&^	6.87±4.1***^,&&&^	43.31±11.9
Sperm total motility (%)	59.38±9.7	23.54±15.4***^,&&&^	17.5±9.6***^,&&&^	58.9±6.9
Sperm normal forms (%)	7.92±0.8	2.17±0.8***^,&&&^	1.83±.7***^,&&&^	6.20±0.7
Seminal calbindin2 (ng/ml)	5.02±0.9	4.37±1.3	3.68±0.1***^,@@@,&&&^	4.04±1.2

**,*** p < 0.01, p < 0.001 compared to controls

^@@@^p < 0.001 compared to infertile OAT without varicocele

^##^p < 0.01 compared to infertile OAT with varicocele

^&&,&&&^p < 0.01, p < 0.001 compared to fertile normozoospermic with varicocele

As expected both of the OAT groups had significantly lower sperm concentration than the fertile normozoospermic control group and the fertile normozoospermic with varicocele group. The infertile OAT with varicocele group had a significantly higher percentage of participants with moderately low and severely low sperm concentration compared to the infertile OAT without varicocele group (Table 2).

**Table 2:** Shows percentages of different sperm concentrations among the participants

	Controls (Fertile normozoospermic)		Cases					
			Infertile OAT without varicocele		Infertile OAT with varicocele		Fertile normozoospermic with varicocele	
	N	percentage	N	percentage	N	percentage	N	percentage
Normal to mild low (10-15 x 10^6^/ml)	24	100%	14	58.3%	6	25.0%***	24	100%
Moderate low (5-10 x 10^6^/ml)	0	0	5	20.8%	8	33.3%***	0	0
Severe low (<5 x 10^6^/ml)	0	0	5	20.8%	10	41.7%***	0	0

N = total number of participants in each group

***p< 0.001 compared to infertile OAT without varicocele

The lowest mean of seminal CALB 2 (ng/ml) was found in the patients with severe oligozoospermia. In these participants, the mean level of seminal CALB 2 was significantly lower compared to the participants with moderate and mild oligozoospermia groups (3.33, 4.23, 4.35, respectively) (Table 3).

**Table 3:** Shows correlations between seminal calbindin 2 (CALB 2) and percentages of different sperm concentrations among the participants

	Seminal CALB 2 (ng/ml)			
	*N*	Mean±SD	Minimum	Maximum
Severe low (<5 x 10^6^/ml)	15	3.33±0.79**^,##^	2.50	4.5
Moderate low (5-10 x 10^6^/ml)	13	4.23±0.75	2.50	5.00
Normal to mild low (10-15 x 10^6^/ml)	68	4.35±1.15	3.5	7.00

*N* = total number of participants in each group

**p< 0.01 compared to moderately low sperm concentration

^##^*p* < 0.01 compared to normal to mildly low sperm concentration

The age of the patients with varicocele grade II was significantly higher compared to the patients with varicocele grade I and III (*p*=0.041) (Table 4). Seminal CALB 2 was significantly lower in the patients with varicocele grade III and varicocele grade II compared to the patients with varicocele grade I (*p*=0.001) (Table 4).

**Table 4:** Shows correlation between semen parameters and seminal calbindin 2 (CALB 2) and varicocele (Vx) severity

	Grade I Vx (*N*=8)	Grade II Vx (*N*=18)	Grade III Vx (*N*=22)
	Mean ±SD	Mean ±SD	Mean ±SD
Age (years)	28.88±4.58	31.06±5.89**^,##^	26.91±4.24
Semen volume (ml)	2.83±0.97	3.50±1.08	3.51±0.85
Sperm concentration (106/ml)	22.94±22.78	33.25±23.39	20.47±18.11
Sperm total motility (%)	35.00±22.52	44.17±23.34	34.55±21.82
Sperm normal forms (%)	4.13±2.90	4.67±2.30	3.46±2.06
Seminal CALB 2 (ng/ml)	5.19±1.25	3.89±0.56	3.36±0.71***^,&&&^

*N* = total number of participants in each group

**,*** *p* < 0.01, *p* < 0.001 compared to Grade I Vx.

^&&&^*p* < 0.001 compared to Grade II Vx

^##^*p* < 0.01 compared to Grade III Vx

There were significant differences in sperm concentration, sperm motility, and seminal CALB 2 in participants with unilateral Vx compared to patients with bilateral Vx (*p*=0.008, *p*=0.001, *p*=0.001, respectively) (Table 5).

**Table 5:** Shows the relationship between Vx laterality and semen parameters and seminal calbindin 2 (CALB 2)

	Unilateral Vx (*N*=21)		Bilateral Vx (*N*=27)		P-Value
	Mean	SD	Mean	SD	
Age (years)	28.66	±5.60	28.88	±5.03	0.695
Semen volume (ml)	3.33	±0.10	3.34	±0.97	0.340
Sperm concentration (10^6^/ml)	40.80	±20.77	23.07	±22.58	0.008**
Sperm total motility (%)	44.05	±17.22	24.44	±15.59	0.001***
Sperm normal forms (%)	4.19	±0.18	3.37	±0.90	0.172
Seminal CALB 2 (ng/ml)	3.90	±0.49	5.31	±0.39	0.001***

**Fig. 1:**
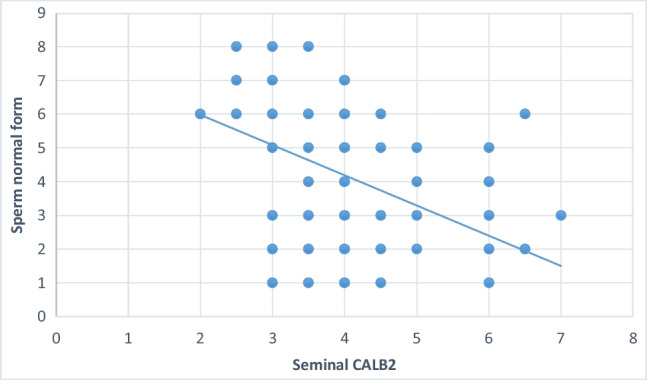
Shows the correlation between seminal plasma CALB 2 and percentage of sperm normal forms

**Fig. 2:**
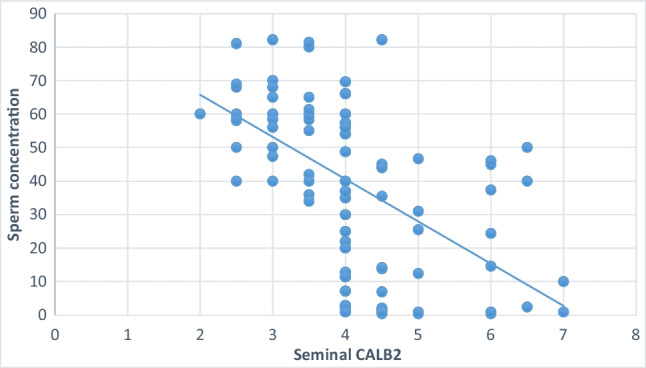
Shows the correlation between seminal plasma CALB 2 and sperm concentration

**Fig. 3:**
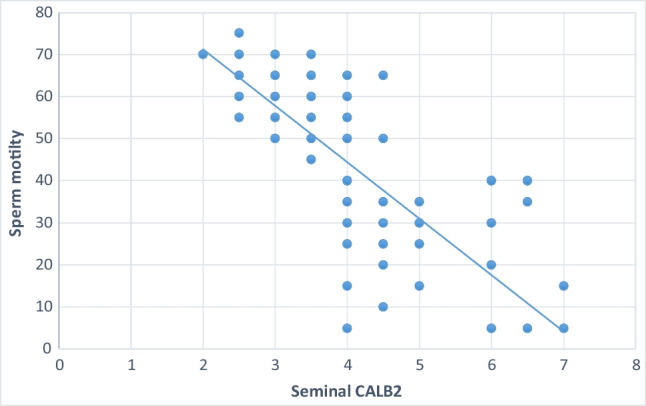
Shows the correlation between seminal plasma CALB 2 and sperm motility

Significant negative correlations were noted between sperm concentration, sperm motility and percentage of sperm normal forms and seminal CALB 2 (Table 6, Figures 1, 2, and 3).

**Table 6:** Shows correlation between seminal CALB 2 Level and semen parameters

		Seminal CALB 2 (ng/ml)
Age	pearson correlation (r)	-0.041
*p*-value	0.689	
Semen volume (ml)	pearson correlation (r)	-0.046
*p*-value	0.657	
Sperm concentration (10^6^/ml)	pearson correlation (r)	-0.589
*p*-value	0.001***	
Sperm total motility (%)	pearson correlation (r)	-0.760
*p*-value	0.001***	
Sperm normal forms (%)	pearson correlation (r)	-0.426
	*p*-value	0.001***

## Discussion

The current study was a case control study conducted over a period of 6 months. The total number of participants was 96 divided into 4 equal groups: Group I, controls (fertile normozoospermic men without varicocele) (*n*=24); Group (II), fertile normozoospermic men with varicocele (*n*=24); Group (III), infertile oligoasthenoteratozoospermia (OAT) men without varicocele (*n*=24); and Group (IV), infertile OAT men with varicocele (*n*=24). There was a significant statistical difference between the studied groups regarding sperm concentration, sperm total motility, percentage of sperm normal forms and seminal CALB 2. As expected, sperm concentration, motility, and percent normal forms was significantly lower in the two infertile groups compared to the fertile normozoospermic groups (Table 1). However, there was no difference in these parameters in the control fertile normozoospermic group (without varicocele) compared to the fertile normozoospermic with varicocele group or between the infertile OAT without varicocele group and the infertile OAT with varicocele group. The percentage of men with severe low sperm concentration was significantly higher in the OAT with varicocele group (41.7%) than in the OAT without varicocele group (20.8%) or in the fertile groups (0%). However, there were no correlations between semen parameters and increasing grade of varicocele, as shown in Table 4.

Remarkably, around 25% to 40% of men with semen alterations had varicocele, yet, the exact mechanism by which this condition impairs sperm quality remains unclear [26]. Furthermore, most men with a varicocele can father children and the exact link between varicoceles and infertility is still questionable [9]. As can be seen in Table 2, 24 patients in the fertile with normozoospermic semen with varicocele group and 6 patients in the infertile with varicocele group had optimal semen quality from a fecundity perspective. Thus, the percentage of patients with varicocele but with optimal semen quality compared to all patients with optimal semen quality was 30/68 (41.1%). This is higher than the percentage of volunteers in the general population from Barcelona with normal sperm counts and normal sperm motility (22%) and in the general Danish population with normal sperm counts and normal sperm motility (23%) [27, 28]. These results, in agreement with the results summarized above, do not support an association between varicocele and abnormal sperm count or motility. Seminal CALB 2 levels were significantly lower in the infertile OAT with varicocele group compared to the other 3 groups (Table 1). Seminal CALB 2 levels were also lower, although without statistical significance, in the fertile normozoospermic with varicocele group compared to the two groups without varicocele, the fertile normozoospermic without varicocele and the infertile OAT without varicocele groups. In addition, seminal CALB 2 decreased with increasing grade of varicocele (Table 4). These data suggest an association between CALB 2 levels and varicocele. CALB 2 levels were significantly lower in men with severely low sperm concentration compared to men with moderately low or mildly low to normal sperm concentration (Table 3). In addition, there was a significant decline of sperm concentration, motility, and percent normal forms with decreasing seminal CALB 2 levels (Table 6). These data suggest an association between CALB 2 levels and sperm quality. Table 5 shows semen volume, sperm concentration, sperm motility, percent sperm normal forms, and seminal CALB 2 in patients with unilateral varicocele and bilateral varicocele. In patients with bilateral varicocele, seminal CALB 2 levels were significantly higher than in the patients with unilateral varicocele, but sperm concentration and motility were significantly decreased compared to the patients with unilateral varicocele. These results are in apparent contrast with the results summarized above. However, they are in agreement with another study in which CALB 2 was expressed in immature Sertoli cells in patients with non-obstructive azoospermia [23].

The aforementioned study concluded that there was a functional relationship between CALB 2 expression and Sertoli cell differentiation, which is in agreement with our results. Patients with bilateral varicocele would have more immature Sertoli cells compared to patients with unilateral varicocele and consequently have increased levels of CALB 2 expression by the seminiferous epithelium and consequently have increased levels of seminal CALB 2 compared to patients with unilateral varicocele. At the same time, patients with bilateral varicocele would have fewer mature Sertoli cells and consequently have decreased sperm production compared to patients with unilateral varicocele. These results also suggest that production of CALB 2 by cells other than Sertoli cells may be required for optimal sperm quality. We found that the mean age was significantly higher in the infertile OAT without varicocele group compared to the other three groups. However, the age difference was relatively small and its significance, if any, remains to be determined. Literature on the impact of paternal age on semen parameters remains inconclusive [29]. It is becoming increasingly important to determine whether advanced paternal age is associated with diminished semen quality and a higher risk of infertility as couples delay childbearing [30]. A retrospective analysis reported that sperm concentration declined after 40 years of age and sperm motility decreased after 43 years of age [31]. Pasqualotto et al (2018) identified age thresholds of 45 years for declining sperm concentration and motility [32]. Sloter et al (2016) also found a correlation between decreased sperm motility with increasing age [33]. Admittedly, we recognized several limitations of the current study. This study is an initial study. Thus, it is reasonable that the number of study participants is not large and that CALB 2 was not measured in patients after varicocelectomy. Furthermore, the present study measured a single protein, CALB 2, in the seminal plasma which cannot be considered a proteomic analysis of the semen sample. However, studies analyzing the proteomic content of semen samples from infertile patients are lacking [34]. Finally, this study paves the way for larger more complex studies of CALB 2 levels (and possibly additional protein analysis) in the future.

## Conclusion

To the best of our knowledge, the current study is one of the first to evaluate the seminal level of CALB 2 among patients with varicocele. The study has demonstrated a potential interplay between CALB 2 and varicocele. Decreased production of seminal plasma CALB 2 also appeared to have a negative impact on sperm concentration, sperm motility, and the percentage of sperm normal forms. Taken together, our data are consistent with varicocele having a negative impact on sperm quality, and seminal CALB 2 may play a role in the negative impact of varicocele on sperm quality. Finally, production of CALB 2 by cells other than Sertoli cells appears to be required for optimal sperm quality. Future studies are needed to verify these findings.

## Data Availability

All authors can show the data of the study upon reasonable request from the editorial board of the journal.

## Code Availability

(SPSS version 25 (Statistical package for social science) for windows 10).
